# Conflict Background Triggered Congruency Sequence Effects in Graphic Judgment Task

**DOI:** 10.1371/journal.pone.0054780

**Published:** 2013-01-23

**Authors:** Liang Zhao, Yonghui Wang

**Affiliations:** 1 School of Psychology, Shaanxi Normal University, Xi’an, China; 2 Shaanxi Provincial Key Laboratory of Behavior and Cognitive Neuroscience, Xi’an, China; University of Missouri-Kansas City, United States of America

## Abstract

Congruency sequence effects refer to the reduction of congruency effects when following an incongruent trial than following a congruent trial. The conflict monitoring account, one of the most influential contributions to this effect, assumes that the sequential modulations are evoked by response conflict. The present study aimed at exploring the congruency sequence effects in the absence of response conflict. We found congruency sequence effects occurred in graphic judgment task, in which the conflict stimuli acted as irrelevant information. The findings reveal that processing task-irrelevant conflict stimulus features could also induce sequential modulations of interference. The results do not support the interpretation of conflict monitoring and favor a feature integration account that the congruency sequence effects are attributed to the repetitions of stimulus and response features.

## Introduction

Recent studies have demonstrated that the congruency effects would reduce when following an incongruent trial than following a congruent trial in a Stroop like task. The decline in performance on incongruent relative to congruent trials is termed congruency effects (also known as conflict effects). For example, when the participants are asked to name the color of a word which is semantically incongruent with its meaning (e.g., printed in green when the word is “RED”), the response time will be longer than that of the color and the word’s meaning are semantically congruent (e.g., printed in green when the word is also “GREEN”) [1], [2]. However, this congruency effects are strongly modulated by the congruency of the previous trial: when the previous trial is incongruent, the congruency effects will be weaker than that is congruent, which is termed congruency sequence effects [3]. The congruency sequence effects have been observed across diverse tasks, such as the Simon task [4], [5], [6], [7], [8], Stroop task [9], [10], [11], [12], [13], and flanker task [14], [15], [16], [17], [18]. One of the most influential contributions to this research area is the conflict monitoring theory [19], [20]. According to this account, changes in congruency effects are truly caused by fluctuations in cognitive control. When competing responses (or response conflict) is simultaneously activated on an incongruent trial, the conflict between responses is detected by the anterior cingulate cortex (ACC). The detection of such conflict acts as a signal for other brain areas, in particular the lateral prefrontal cortex (LPFC), which subsequently engages in recruiting control processes to overcome the conflict. The increase in cognitive control results in the higher processing of the relevant stimulus dimension relative to the irrelevant dimension. This account has been supported by some researches appeared to reflect online reactive adjustments in control [6], [9], [10], [11], [20], [21], [22], [23], [24].

Besides, some researchers argue that the congruency sequence effects can be attributed to the repetitions of stimulus and response features (feature integration) without top-down regulation [25], [26]. The basic idea is that when stimuli and responses co-occur in time, their features are spontaneously encoded into a short-lived episodic memory representation, which is called an event file [27]. On any given trial, the stimulus and response features are temporarily associated with each other. When some but not all features of the stimulus–response episode violates this association on the next trial, reaction times (RTs) tend to be longer, since the previous feature binding has to be overcome. While every feature is repeated or alternated, responding is faster, because no previous feature binding has to be overcome. This hypothesis has also been supported by some empirical evidence [16], [25], [26], [28].

To test these two alternative hypotheses, most researchers adopted conflict tasks and advocated the use of only those conditions that were unaffected by feature integration, where neither the stimulus feature nor the response feature was repeated. Therefore, any observed congruency sequence effects hereby should be attributed to cognitive control process. Whereas, if none of the congruency sequence effects were observed, this sequential modulation would be attributed to feature integration. Unfortunately, previous studies did not reach a unified conclusion.

In the present study, we sidestepped the conventional methods by using a graphic judgment task to explore the congruency sequence effects, in which conflict stimuli constituted irrelevant information, i.e., there was a Stroop color word in the center of each graphics, and the participants were required to respond to graphic shapes (triangle, square, rhombus, or pentagon). There were two advantages in this task. First, the irrelevant information (conflict stimuli) was either congruent (ink color and word meaning are semantically congruent) or incongruent (ink color and word meaning are semantically incongruent), which could be taken as independent variables to explore the congruency sequence effects. Second, in the present graphic judgment task, there were no competing responses between graph and Stroop stimuli, which was deviant from the former research paradigms concerning sequential modulation. To be emphasized that an essential aspect of conflict monitoring theory is how the conflict is generated. On the basis of this account, conflict is defined as the simultaneous activation of competing responses. When two response candidates (e.g., left response and right response) are both activated, response conflict occurs and is detected by ACC, leading to the adjustments in cognitive control. This was supported by some studies. For example, some researchers [29], [30] had demonstrated that the ACC was activated only when a response conflict was detected, moreover, the conflict was evoked by the joint activation of two different responses. However, in the present task, the Stroop stimuli were only the irrelevant information presented in the center of the graphic, which did not constitute competing responses when the participants were making the graphic judgment. This manipulation did not comply with the premise of the conflict monitoring theory. Thus, if congruency sequence effects are found in this task, it will demonstrate that the conflict monitoring is not the only mechanism to trigger the congruency sequence effects, and feature integration is an additional alternative.

## Methods

### Ethics Statement

All procedures were executed in compliance with relevant laws and institutional guidelines and were approved by the ethics committee of the Psychology school of Shaanxi Normal University. The participants provided informed consent prior to the experiment.

### Participants

13 female and 11 male students from Shaanxi Normal University participated in the experiment in exchange for partial course requirements or on a voluntary basis. They ranged in age from 21 to 26 years.

### Apparatus and Stimuli

Stimuli were presented on a 17-in. monitor with a refresh rate of 60 Hz which placed at a distance of 60 cm from the participant. The stimuli were consisted of graphics with Stroop words inside. Graphics involved triangle, square, rhombus, and pentagon. The Stroop words, including the Chinese characters RED or GREEN in red or green ink, were embedded in the central of graphics. Each of the graphical stimuli (one graphics with one character inside) using in the experiment was approximately 3.3° tall and 2.3° wide, and the font size of the character was 1.9° tall and 1.9° wide. They were presented at the center of computer screen with gray background. The participants had to judge the graphic shapes by pressing the corresponding key on the keyboard. For one half of the participants, triangle and rhombus were mapped onto the left response key (A key), whereas square and pentagon were mapped onto the right response key (L key). For the remainder of the participants this assignment was reversed. Here, graphics with congruent color words were termed congruent trials, and that with incongruent words were termed incongruent trials. According to the congruency on previous trial and current trial, the trials can be divided into four types: CC trials (congruent stimuli following a congruent stimulus), CI trials (incongruent stimuli following a congruent stimulus), IC trials (congruent stimuli following an incongruent stimulus), and II trials (incongruent stimuli following an incongruent stimulus).

### Procedure

Each block began with the presentation of a fixation cross in the center of the screen (1.1°×1.1°). After a period of 1,000 ms, the fixation disappeared and stimuli appeared. The participants were instructed to respond to the graphic shapes as quickly as possible while avoiding errors. Immediately after a response key was pressed, stimuli disappeared from the screen. The display presented for a maximum of 2,000 ms. Following a 1,000-ms blank interval, the next trial started.

Stimuli were arranged with pseudo randomized order, which premade separately for each block to ensure each stimulus combination sequence was equal, thus resulting in an equal proportion of CC, CI, IC, and II trials. In each block, the numbers of responses either by left or right hand were balanced, and were guaranteed no more than three same reactions continuously. The participants received 33 practice trials before entering the experimental phase, which consisted of 8 blocks of 65 trials each. In the experimental phase, they were allowed to rest for some time between blocks.

## Results

### RTs

We excluded the first trial of each block, errors, trials following an error, and condition-specific outlier values (>2.5 SDs from the mean), which constituted 7.30% of all the trials. A two-way analysis of variance (ANOVA) was conducted on the RTs with the following variables: congruency on previous trial (congruent vs. incongruent) and congruency on current trial (congruent vs. incongruent).

**Figure 1:**
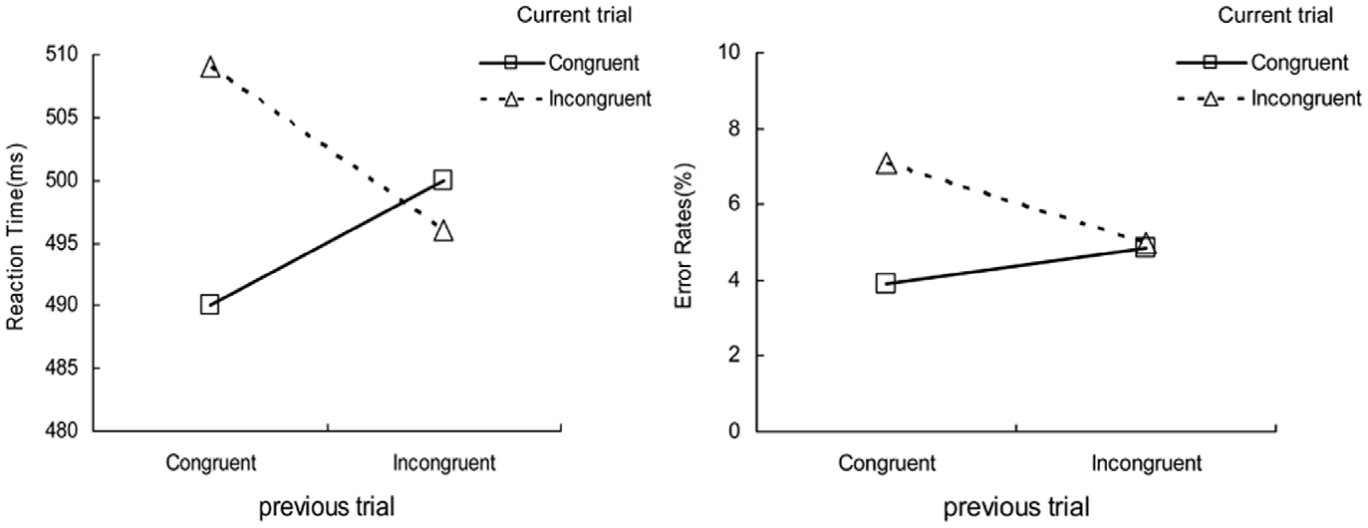
Congruency sequence effects. Mean reaction times (RTs) and mean error rates for congruent and incongruent trials in the present trial (N) depending on the congruency of the previous trial (N−1).

As shown in Figure 1 (left panel), both the main effects of congruency on previous trial, *F* (1, 23) = 0.27, *p = *.609, and congruency on current trial, *F* (1, 23) = 1.84, *p = *1.888, were not significant. However, the interaction between congruency on previous trial and current trial was significant, *F* (1, 23) = 6.52, *p*<.05, indicating the typical congruency sequence effects: congruency effects were reduced after incongruent trials (−4.45 ms) compared with congruent ones (18.58 ms).

### Errors

The first trials of each block were excluded, because we were examining sequential effects. A same two-way ANOVA analysis was conducted as the RT’s. As depicted in Figure 1 (right panel), there was a significant main effect of current trial congruency, *F* (1, 23) = 6.04, *p*<.05, with being more errors for incongruent trials (6.02%) than for congruent trials (4.35%). The congruency on previous trial did not reach significance, *F* (1, 23) = 0.39, *p = *.537. More importantly, there was a significant interaction between the congruency of previous trial and current trial, *F* (1, 23) = 5.32, *p*<.05. Further analyses showed that congruency effects after incongruent trials (0.13%) were smaller than after congruent trials (3.21%).

The analyses above suggested that the congruency sequence effects were triggered by the task-irrelevant conflict stimulus in the absence of response conflict. To analyze the alternative repetition-effect, we re-analyzed the data that followed the logic of the integration approach suggested by Hommel [27] and Hommel et al. [25]. Accordingly, mean RTs and mean error rates were analyzed as a function of the repetition versus alternation of response key (here confounded with graphic shape) and Stroop stimulus congruency level. However, in the current design, four possible graphic values were mapped onto two responses, resulting in a particular condition that the graphic shape features change (from e.g. triangle to rhombus) while the response features stay the same, which was suitable neither for repetition of response key (confounded with graphic shape) nor for alternation of response key (confounded with graphic shape). Thus, these trials were not included in the analysis.

**Figure 2:**
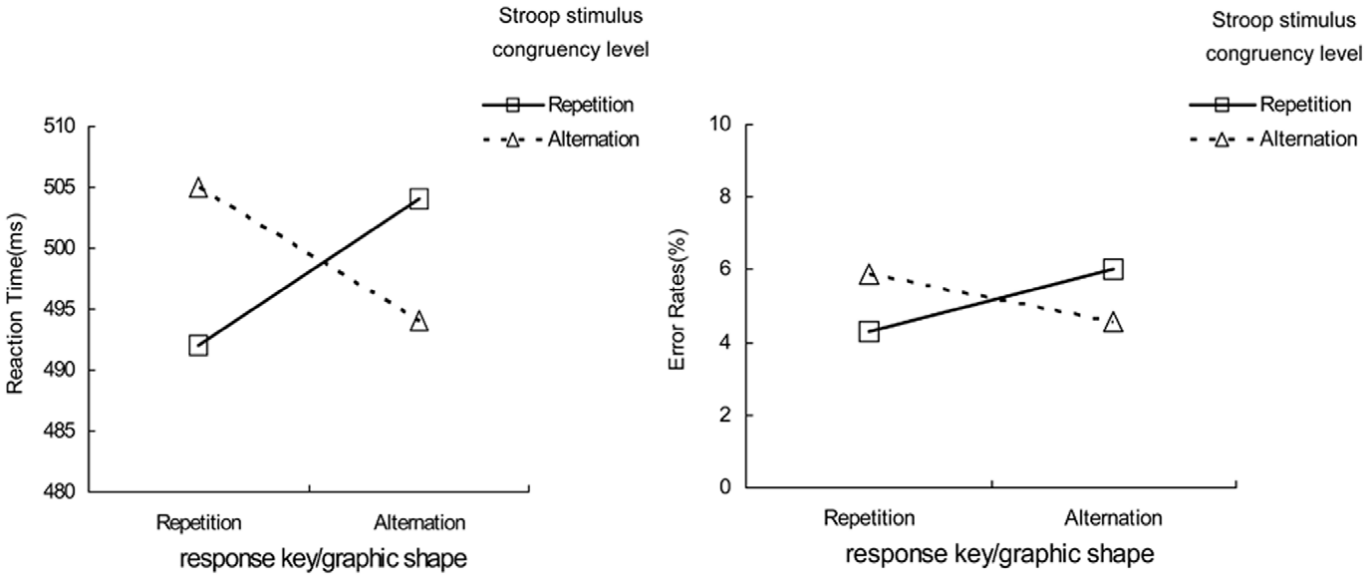
Feature integration effects. Mean reaction times (RTs) and mean error rates as a function of the repetition versus alternation of response key/graphic shape and Stroop stimulus congruency level.

For the RTs (see Figure 2, left panel), the main repetition effects of response key/graphic shape and Stroop stimulus congruency level (*all Fs*<1) were not significant. However, the interaction of response key/graphic shape and Stroop stimulus congruency level was significant, *F* (1, 23) = 6.85, *p*<.05. RTs were fast if response key/graphic shape and Stroop stimulus congruency level were both repeated (492 ms) or both alternated (494 ms), but slow if only response key/graphic shape (505 ms) or only Stroop stimulus congruency level (504 ms) was repeated while the other feature was alternated. The error rate analysis reached very similar results as those obtained for RTs (see Figure 2, right panel). A significant interaction of response key/graphic shape and Stroop stimulus congruency level, *F* (1, 23) = 5.37, *p*<.05, indicated that error rates decreased if response key/graphic shape and Stroop stimulus congruency level were either both repeated or both alternated (4.28% and 4.56% respectively) than if only one, but not all, was repeated (5.88% and 6.03%, respectively).

A separate analysis was conducted for those trials which were not included in the above analysis. According the logic of feature integration, if some of the features change but others do not, this should lead to slower RTs, since some associations need to be overcome. We separately compared this with every feature repetition or alternation. Results showed that participates responded slower when graphic shape features changed (from e.g. triangle to rhombus) while the other features (same response, same congruency level) stayed the same (492 ms) than every feature repetition (502 ms), *t* (23) = 2.37, *p*<0.05. Results also showed that participates responded slower when graphic shape features alternated (from e.g. triangle to rhombus) and congruency level alternated while the other features (same response) repeated (506 ms) than every feature alternation (494 ms), *t* (23) = 2.42, *p*<0.05.

## Discussion

Previous researches mostly focus on the situation with the conflict stimuli as primary target. In the present study, we explored the congruency sequence effects from a new perspective, in which the conflict stimuli constituted irrelevant information. Specifically, this study was concerned with the performance of graphic shapes judgment, wherein, the Stroop stimuli were embedded inside the graphics acting as the irrelevant information. We found that the RTs difference between graphics with incongruent Stroop stimuli and graphics with congruent Stroop stimuli reduced when following an incongruent trial (graphics with incongruent Stroop stimulus) comparing with a congruent trial (graphics with congruent Stroop stimulus), which manifested congruency sequence effects. The results suggest that processing task-irrelevant conflict stimulus features could also induce sequential modulations of interference.

Note that, this is the first time that the sequential modulations evoked by processing task-irrelevant conflict stimuli have been observed. We consider that the phenomenon occurred in the present study fit well with the feature integration account to a great extent. The results may suggest that the size of interference caused by irrelevant stimuli is possibly modulated by congruency on previous trial.

As previously mentioned, in the former studies, researchers usually adopted conflict tasks, in which competing responses between relevant information and irrelevant information would induce response conflict. However, in this study, none of competing responses could be activated when the participants were making the graphic judgment. Although there was a conflict between the characters red and green and the color of the ink, response needn’t be executed for the Stroop stimuli, resulting in no competing responses between the graph and Stroop stimuli. In another study, Hommel et al. [25] conducted an experiment (Experiment 3) in which no response was executed to the stimulus preceding the target, yet sequential modulations of congruence effects were obtained, which was as evidence in favor of feature integration. The presence of an interaction between response key/graphic shape and Stroop stimulus congruency level in the current study also fit well with the findings of Hommel [27] and Hommel et al.[25].

Obviously, the results of this study could not be explained by the conflict monitoring theory, but were probably compliance with feature integration account to a great extent. The feature integration account assumes that when stimuli and responses co-occur in time, their features are spontaneously encoded into a short-lived episodic memory representation termed event file. In the present experiment, graphic shape features, Stroop stimuli features, and response features can be bound into an event file. The activation of any one of these features will lead to activation of associated features. If some but not all features of the stimulus–response episode violates this association on the next trial, reaction times will be slower, since the repeating features automatically activate other features in the event file from the previous trial and the association has to be overcome. If every feature repeats or alternates, responding will be faster, because no previous feature association has to be overcome.

Due to the restriction of experimental design which adopted a single two-choice task, we performed an analysis that confounded the response key and graphic shape, just as Experiment 1 of Hommel et al. [25]. The possible combination of response key-Stroop stimulus congruency level and graphic shape-Stroop stimulus congruency level integration effects may also make independent contributions. Although two-choice experiments do not allow a full-proof test for feature integration effects, these results suggested that congruency sequence effects can not be fully explained by conflict monitoring. However, further systematic studies need to be conducted to unconfound response key and graphic shape effects.

### Conclusions

In summary, the pattern of results obtained in the present study clearly indicates that irrelevant information, which involves conflict information, can also trigger sequential modulations. This finding corroborates and extends earlier findings by demonstrating that adjustments of cognitive control are not the only possible explanation of congruency sequence effects. Feature integration is an additional mechanism.
